# Successful surgical intervention for rectal perforation due to polyarteritis nodosa: report of a case

**DOI:** 10.1186/s40792-017-0316-0

**Published:** 2017-03-13

**Authors:** Keiji Yoshiya, Yu Imamura, Yu Nakaji, Daisuke Taniguchi, Rinne Takeda, Koji Ando, Yuichiro Nakashima, Motohiro Shimizu, Kippei Ohgaki, Norihiro Furusyo, Takuya Matsumoto, Hiroshi Saeki, Yoshinao Oda, Eiji Oki, Yoshihiko Maehara

**Affiliations:** 1 Department of Surgery and Science, Graduate School of Medical Sciences, Kyushu University, 3-1-1 Maidashi, Higashi-ku, Fukuoka, 812-8582 Fukuoka Japan; 2 Department of Gastroenterological Surgery, the Cancer Institute Hospital of Japanese Foundation of Cancer Research, Tokyo, Japan; 3 Department of Anatomic Pathology, Pathological Sciences, Graduate School of Medical Sciences, Kyushu University, Fukuoka, Japan; 4 Department of General Internal Medicine, Kyushu University Hospital, Fukuoka, Japan

**Keywords:** Polyarteritis nodosa, Rectal perforation, Peritonitis

## Abstract

**Background:**

Polyarteritis nodosa (PAN) is a primary systemic necrotizing vasculitis with diffuse organ involvements, resulting in a high mortality rate due to multiple organ failure. Although the small bowel is the frequently targeted organ of PAN-associated vasculitis, rectal involvement is very rare, and only one case of rectal bleeding has been previously reported. The mortality rate of PAN with gastrointestinal (GI) perforation is reportedly much higher than that of without severe GI involvement. We herein report the first case of rectal perforation due to PAN, successfully managed with an adequate surgical intervention.

**Case presentation:**

A 66-year-old woman with PAN had abdominal pain and melena with guarding. Computed tomography scan showed abdominal free air and bubbles in the rectal hematoma. We diagnosed it acute peritonitis, and emergency surgery was performed. After removing rectal hematoma and necrotic tissue, a huge lack of rectal wall spreading to the pelvirectal space was observed. In order to totally remove the necrotic tissue, abdominoperineal resection was needed. Together with histopathological examinations which showed neutrophils and fibrinous necrosis, we finally diagnosed rectal perforation due to PAN. At 19-month follow-up after surgery, she was still healthy with a stable disease of PAN.

**Conclusions:**

We herein reported the first case of successfully managed rectal perforation due to PAN. Early adequate surgical resection may be important for the case with rectal perforation.

## Background

Polyarteritis nodosa (PAN) is a primary systemic necrotizing vasculitis that predominantly targets medium-sized arteries defined as the main visceral arteries and their branches [1]. Disease spectrum of PAN involves multiple organs [1, 2]. Fibrinoid necrosis leads to microaneurysm, which is frequently observed in renal, cerebral, and coronary vessels, resulting in progressive organ failure due to infarction and hemorrhage. Corticoteroids and immunosuppressants like cyclophosphamide (CPA) are usually recommended for the management of PAN [3].

Around 40% of the patients with PAN has gastrointestinal (GI) involvements [4], commonly in the small bowel, and rectal involvement is quite rare [5]. Severe GI involvements such as bleeding and perforations have been reported to be major factors of poor prognosis [6]. We herein report a case of successful management of the rectal perforation due to PAN, with a surgical intervention of abdominoperineal resection.

## Case presentation

**Fig. 1:**
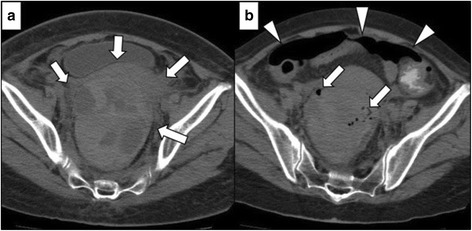
Computed tomography (CT) findings. **a** Rectal hematoma (*arrows*) **b** Intraperitoneal free air (*arrow heads*) and bubbles in the rectal hematoma (*arrows*)

**Fig. 2:**
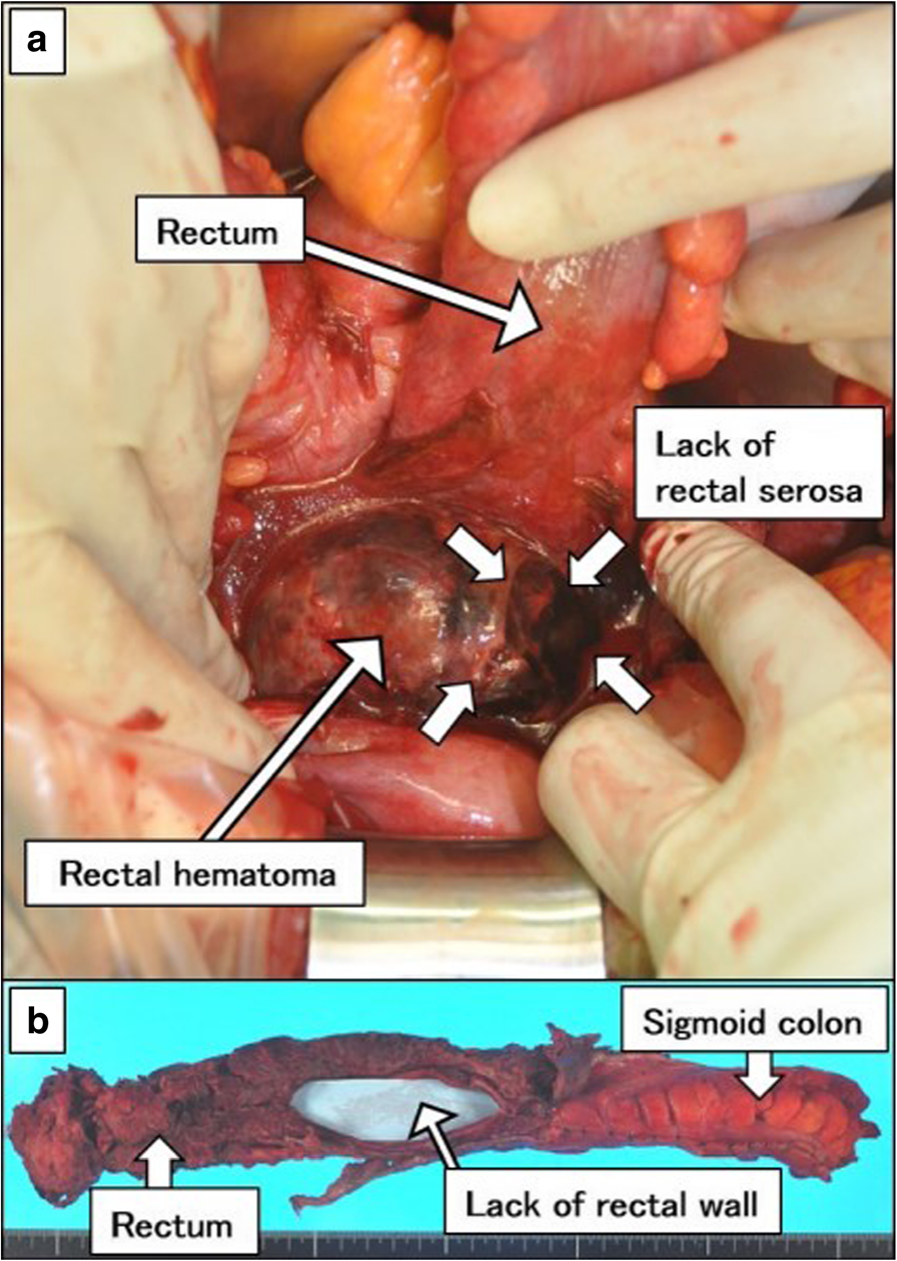
Intraoperative findings. **a** Rectal hematoma with partial defect of rectal serosa. **b** Huge defect of rectal wall (*blue* sheet was placed in the lumen, showing the defect)

A 66-year-old woman with PAN and renal dysfunction was admitted to the Department of General Internal Medicine of our hospital. Pulse steroid therapy (methylprednisolone, 1 g/day, for 3 days) had been administered, followed by 1 mg/kg/day prednisolone (PSL). Four days after the pulse steroid therapy started, she had abdominal pain and melena without guarding. Computed tomography (CT) scan showed high-density areas around the rectal wall (Fig. 1a). We diagnosed hematoma in the rectal wall, and 1 mg/kg/day of CPA was added to steroid therapy. Her abdominal symptom once disappeared, but it recurred with guarding 14 days after the introduction of pulse steroid therapy. Abdominal free air and bubbles in the rectal hematoma were detected on CT scan (Fig. 1b). She was in septic shock with tachycardia, tachypnea, and hypotension. A laboratory examination demonstrated decreased white blood cell count of 7890/uL (24,800/uL, the day before), platelet count of 8.6 × 10^4^/uL and C-reactive protein of 7.31 mg/dL, and serum creatinine of 240 kmol/L. We diagnosed it acute peritonitis, and emergency surgery was performed. Bloody ascites and rectal hematoma with partial lack of rectal serosa were observed (Fig. 2a). Because rectal hematoma and necrotic tissue was extending to the anal, abdominoperineal resection was necessary for this case. There was no evidence of intestinal perforation from the stomach to the sigmoid colon. After removing the rectal hematoma and necrotic tissue, a huge lack of rectal wall spreading to the pelvirectal space was observed (Fig. 2b). Thus, we diagnosed it as rectal perforation due to rectal hematoma and necrosis. After surgery, both PSL (60 mg/day) and CPA (25 mg/day) were needed in order to control PAN. Blood transfusion was performed for anemia caused by CPA. PSL could be tapered down to 25 mg/day on the 90th postoperative day. One hundred days after the operation, she transferred to the outside hospital for further rehabilitation. At 19-month-follow-up after surgery, she was still healthy with a stable disease of PAN using 10 mg/day of PSL.

**Fig. 3:**
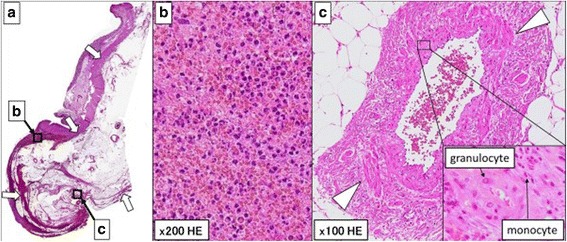
**a**, **b** A mixture of hematoma and abscess (*arrows*) in the rectal wall in hematoxylin-eosin (HE) staining (**a** magnification ×20; **b** magnification ×200). **c** Granulocytes and monocytes (highly magnified image *in a small window*) and fibrinous necrosis (*arrow heads*) in the vessel were observed in HE (magnification ×100)

Histopathological examinations revealed that hematoma and abscess were observed in the rectal wall (Fig. 3a, b). In addition, neutrophils and fibrinous necrosis, which are pathological characteristics of PAN [3], were found in the vessel of the rectum near the region of the perforation (Fig. 3c). Together with the intraoperative findings of rectal perforation due to rectal hematoma and necrosis, we finally diagnosed rectal perforation due to PAN.

### Discussion

PAN is a medium-sized vessel vasculitis disease, involving multiple organs such as the skin, heart, kidneys, and nervous system [1]. GI involvements are generally rare but are clinically important because of their serious complications of perforation and infarction [6]. Some previous reports showed a high rate of mortality in PAN, due to those acute abdominal complications. GI involvements of PAN patients commonly occur in the intestine from the small bowel to the colon [4, 7]. Rectal involvements are quite rare, and there is one case report of rectal hemorrhage [8], but no rectal perforation. To the best our knowledge, this is the first case of rectal perforation due to PAN.

Pagnoux and his colleagues showed six cases (15.8%, among 38 patients with PAN) presented bowel perforation, which was one of the significant prognostic factor of PAN (HR = 5.7, *p* < 0.01), though the sites of perforation were not specified. Therefore, prediction of bowel perforation may be clinically important in the management of PAN [4]. Five-Factor Score (FFS) for systemic necrotizing vasculitis has been used to evaluate prognosis at diagnosis since 1996. In 2009, FFS parameters were revised as follows: age >65 years, cardiac insufficiency, renal insufficiency (stabilized peak creatinine ≧150 kmol/L), and severe GI involvement (bowel perforation, bleeding, and pancreatitis, with neither appendicitis nor cholecystitis) and absence of ear, nose, and throat involvement. These clinical symptoms comprising the score were directly attributable to active vasculitis. According to the 2009 FFS, 5-year mortality rates for scores of 0, 1, and ≧2 were 9, 21, and 40%, respectively [9]. Bourgarit et al. reported that uncontrolled vasculitis and infection were the major causes of early death in PAN patients. In addition, insufficient therapy for PAN results in fatal disease [10]. Despite that our case scored 3 of FFS and was resistant to pulse steroid therapy, we successfully managed this case with an adequate surgical intervention. Early diagnosis of peritonitis, early surgical intervention, and quick decision to totally remove necrotic tissue during surgery enabled us to get control of the infection and resumed treating PAN with adequate immunosuppressive medicines early after the rectal perforation. Those were the key for successful management of this case with PAN.

## Conclusions

We herein reported the first case of successfully managed rectal perforation due to PAN. Early adequate surgical resection may be important for the case with rectal perforation.

## Abbreviations

CPA: Cyclophosphamide
CT: Computed tomography
FFS: Five-Factor Score
GI: Gastrointestinal
PAN: Polyarteritis nodosa
PSL: Prednisolone
